# Ligands-Coordinated Zr-Based MOF for Wastewater Treatment

**DOI:** 10.3390/nano8090655

**Published:** 2018-08-24

**Authors:** Xue-Qing Zhan, Fang-Chang Tsai, Lei Xie, Ke-Deng Zhang, Huan-Li Liu, Ning Ma, Dean Shi, Tao Jiang

**Affiliations:** 1 Hubei Key Laboratory of Polymer Materials, Key Laboratory for the Green Preparation and Application of Functional Materials (Ministry of Education), Hubei Collaborative Innovation Center for Advanced Organic Chemical Materials, School of Materials Science and Engineering, Hubei University, Wuhan 430062, China; m18272163856@163.com (X.-Q.Z); raytse526@163.com (L.X.); zkd19910705@hotmail.com (K.-D.Z.); pangchoudan521@163.com (H.-L.L); jiangtao@hubu.edu.cn (T.J.); 2 Department of Chemistry, Wuhan University, Wuhan 430072, China; maning@whu.edu.cn

**Keywords:** Zr-MOF, adsorption, dye, NLDFT, BET

## Abstract

Isostructural zirconium-based metal–organic frameworks (Zr-MOFs) have attracted the attention of researchers because of their remarkable stability at high temperatures and high pressures and their chemical stabilities against acids and bases. Due to this stability, Zr-MOFs can be utilized in adsorption research, and the adsorption performance of a Zr-MOF depends on the pore size and the surroundings of the MOF. In this study, as the dimensions changed and the adsorption was carried out, the Zr-MOF material remained stable, and the adsorption of the best state was achieved at 235 mg/g. Through the simulation of theoretical kinetic models of Zr-MOFs, we initially postulated that the adsorption capacity is proportional to the pore size and that acid orange 7 (AO7) was adsorbed by the MOFs. Afterwards, we verified our hypotheses through a series of Brunauer–Emmett–Teller (BET) data analysis; non-local density function theory (NLDFT) was mainly used to analyze the data. Moreover, we determined that physical adsorption occurs on the surface of the MOFs during the adsorption process, while chemisorption occurs in the form of dye molecules combining with active sites. Ultimately, we concluded that the larger the pore size, the stronger the adsorption capacity, and this contribution casts a new light on the issue of wastewater treatment.

**Highlights:**

- We have regulated and synthesized different Zr-MOFs with distinct pore sizes by utilizing different lengths of organic ligands.
- The process and mechanisms of the adsorption of dye by Zr-MOFs with different apertures were studied through theoretical kinetic models and BET data analyses.
- The adsorption of the best state was achieved at 235 mg/g.

## 1. Introduction

The disposal of dye-contaminated wastewater, which is produced by the industrial fabrication of textiles, paints, printed materials, pharmaceuticals, food, hair dyes, leather, electronics, cosmetics, and so on, is one of the most pressing global challenges [1,2,3,4]. The contaminants are divided into two categories: natural dyes and synthetic dyes. Azo dyes occupy the greatest proportion of synthetic dyes. Acid orange 7 (AO7) [5] is an example of an azo dye, the harm of which is obvious, and its main harmful effects are enumerated as follows [6]. (I) AO7 endangers human existence and health; for example, in the absence of oxygen, azo dyes can undergo degradation and produce highly toxic aromatic amines, the prolonged exposure to which can lead to apoptosis or trigger genetic mutations. (II) It imparts damage to the appearance of water [7]; when it is dissolved, the dye stains water, and the resulting dark-colored wastewater is difficult to completely decolorize by normal biochemical methods. (III) AO7 affects the survival of aquatic plants and animals; the dye absorbs most of the light penetrating the wastewater, which affects the normal growth of photosynthetic plants and microorganisms in the water. (IV) When AO7 wastewater is discharged directly into open water, it not only leads to the destruction of the water’s ecological system, but its interaction with the pollutants present in the wastewater could lead to the formation of poisonous gases or organic small molecules in aerobic or anaerobic conditions that may cause the destruction of the ecological environment. Numerous studies have been conducted to investigate treatments for dye-contaminated wastewater. For example, membrane separation, adsorption, chemical coagulation, activated sludge, ion exchange, electrochemical methods, biodegradation, oxidation, and photodegradation have been extensively explored [6]. Due to the immense volume of aromatic structures in azo dyes, light and biodegradation, thermal decomposition, and ozonation, as well as oxidation, have been shown to be effective means for treating such dyes [8]. Among all, adsorption has become the typical treatment method.

Adsorptive separation is a subject of interest for the treatment of wastewater, because the process is relatively easy to operate and economical, and at the same time, it does not produce much secondary pollution. As a traditional adsorbent, active carbon had been used widely in the adsorption treatment of dye-contaminated wastewater, which has low capacity, weak interaction, and difficulty of regeneration in practical applications [9,10,11]. Metal–organic frameworks (MOFs) [12] have emerged as a promising platform to prepare adsorbents with desirable adsorption characteristics [13,14]. The structure of a MOF can be modulated by employing various organic bridge or metal ions to achieve the targeted pore size and adsorption characteristics. Moreover, directional control, design, and modification and the regulation of its structure and properties can be realized by changing the organic ligands in the coordination process [15,16]. Familiar multidentate ligands are multicomponent organic carboxylic acids (aliphatic carboxylic acids and aromatic carboxylic acids) and partial organic nitric heterocyclic compounds (pyridine, imidazole, pyrazole, etc.) [17,18,19,20]. Therefore, the various functionalities of MOFs have led to a wide range of applications, including water adsorption [21,22], toxic gas removal [23], gas adsorption and storage [24,25,26], separation [27,28], chemical sensing [29,30], catalysis [31], and energy storage [32]. Recently, zirconium-based MOFs (Zr-MOFs) known as UiOs (University of Oslo) have shown excellent chemical and thermal stabilities toward moisture, as well as toward acids, and have demonstrated high structural stability under high mechanical pressures compared with other types of MOFs [33,34,35]. Because of their structure, the thermostability of UiOs can reach up to 500 °C and stay stable in multiple organic solvents, which is one of the best demonstrated stabilities in the series of MOFs [36]. Good stability makes the material suitable for separation. In the study of MOFs, the modification of pore size through the manipulation of the structure of the inorganic secondary structure units with different lengths of ligands is currently popular [37]. In this respect, the use of longer ligands can reduce the density of the material and increase the surface area [38], and organic ligands with functional groups can promote various intermolecular interactions with molecular or ionic guests [15].

**Figure 1:**
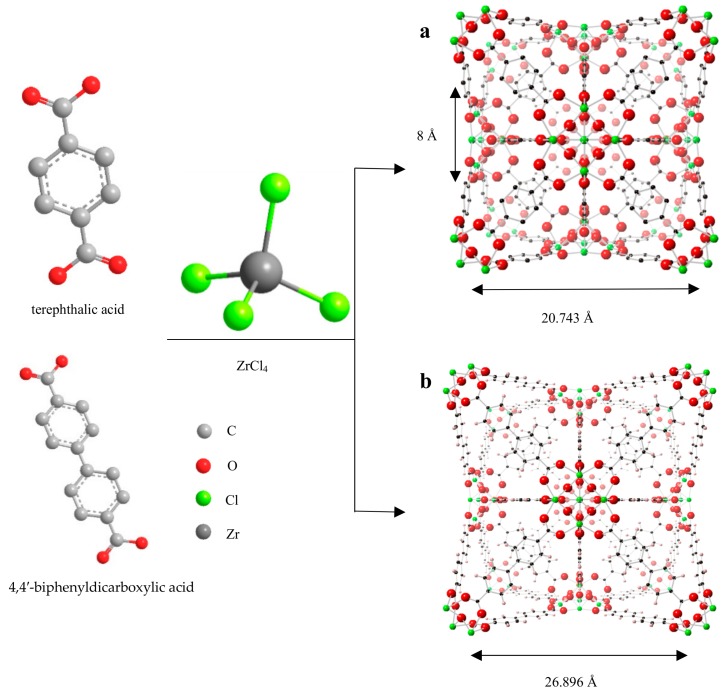
Fabrication of the UiOs. The structure of (**a**) UiO-66, (**b**) UiO-67.

In this work, we have modulated different UiOs with different pore sizes for wastewater treatment. The work presented herein initially aimed to enhance the adsorption capacity of Zr-MOFs by adjusting the lengths of ligands following the aforementioned strategy using terephthalic acid and 4, 4′-biphenyl dicarboxylic acid to synthesize UiO-66 and UiO-67 [39]. UiO-66 and UiO-67 are based on an octahedral Zr_6_O_4_(OH)_4_ cluster, with metal zirconium atoms at the center of 12 junctions connected through the ligand to form a positive tetrahedron and two octahedral structures [33]. The cage structures of the UiOs are shown in Figure 1. The synthesis of the materials occurred in a high-temperature reaction kettle by solvothermal synthesis. In the reaction system, the reaction condition and speed must be considered. HCl, which speeds up the reaction kinetics, may also cause the formation of inherent defects either from misconnections or dislocations during crystallization or from post-crystallization cleavage [40,41]. As reported, the formation of UiO-66 is expected to form a chemical clock [42]: thus, increasing the concentration of the monocarboxylic species carries the same effect as increasing the temperature generally. In this work, the only monocarboxylic species in the synthesis of UiO-66 and UiO-67 that could serve such a role was HCl. The addition of HCl makes the rate of the reaction speed up, which makes the reaction temperature decrease.

**Figure 2:**
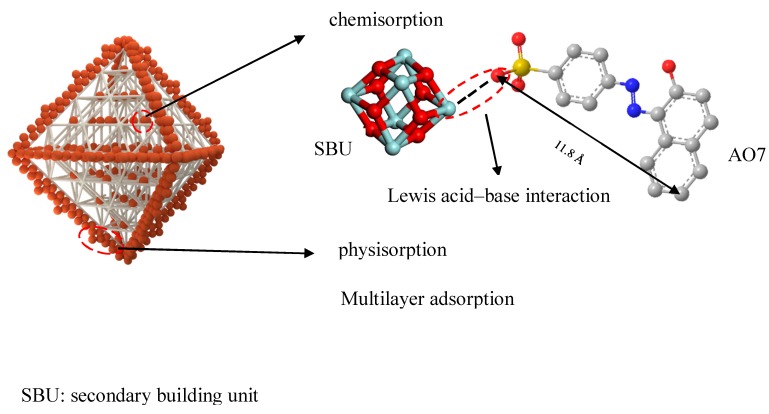
The model of zirconium-based metal-organic frameworks (Zr-MOF) adsorption of acid orange 7 (AO7).

Furthermore, the processing capacity of AO7 in water was further studied. At the same time, adsorption isotherm and adsorption kinetics were analyzed, the adsorption behavior of the UiOs on AO7 was studied, and the adsorption mechanism was assumed (Figure 2). 

## 2. Materials and Methods

### 2.1. Chemicals

All the chemicals and reagents used were of analytical grade. Zirconium (IV) chloride (ZrCl_4_) was purchased from Aladdin (Aladdin Industrial Cooperation, Shanghai, China). 4,4′-biphenyldicarboxylic acid was purchased from TCI Development Co., Ltd. (Shanghai, China) Terephthalic acid, *N*,*N*-dimethylformamide (DMF), and orange II sodium salt were purchased from Sinopharm Chemical Reagent Co., Ltd. (Shanghai, China). Hydrochloric acid was purchased from Xinyang chemical reagent factory (Shanghai, China). 

### 2.2. Synthesis of UiO-66 and UiO-67

The UiO materials were synthesized using a modified method reported previously [43]. Specifically, ZrCl_4_ (0.54 mmol) was dispersed in DMF (5 mL) in an ultrasound, with the addition of HCl (1 mL), and terephthalic acid (0.75 mmol) was dissolved in DMF (10 mL). The metal ions were combined with the ligand under an ultrasound. Then, the mixture was kept at 80 °C for 24 h. The precipitation was washed 3 times with DMF and ethanol (EtOH), respectively, and then dried in a vacuum at 90 °C. However, the synthesis of UiO-67 was quite different. ZrCl_4_ (0.27 mmol) was dispersed in DMF (5 mL) in an ultrasound, with the addition of HCl (0.5 mL), and 4,4′-Biphenyldicarboxylic (0.38 mmol) was dissolved in DMF (15 mL) under a water bath at 80 °C. The metal ions were combined with the ligand under an ultrasound. Then, the mixture was heated to 80 °C and kept for 24 h. The product was washed 3 times with DMF and EtOH, respectively. DMF was put in a water bath at 80 °C. Then, the sample was dried in a vacuum at 90 °C.

### 2.3. Characterization

Powder X-ray diffraction (PXRD) patterns were obtained using a Bruker D8 focus diffractometer (Bruker, Billerica, MA, USA). The measurements were made over a range of 3° < 2*θ* < 40° with a step size of 0.02 at a scanning rate of 10°/min.

A V-Sorb 2800 TP surface area and pore size analyzer (GAPP, Beijing, China) was used to measure the N_2_ adsorption–desorption isotherms of samples at 77 K.

The absorbance measurements of all the AO7 aqueous solutions were performed on a UV–visible spectrophotometer (TU-1810DSPC, Purkinje General Instrument Co., Ltd., Beijing, China).

### 2.4. Adsorption Experiment

The adsorption experiments were executed at 37 °C in a thermostat water bath. The samples of UiO-66 and UiO-67 (10 mg) were respectively added to AO7 solutions with concentration gradients of 10, 20, 30, 40, and 50 ppm (50 mL) to explore the relationship between adsorption capacity and time. The absorbance was measured by UV-Vis spectroscopy at 484.5 nm wavelengths. Then, the amount of the absorbed dye, *q_t_* (mg/g), was calculated by the following equation [44]:

(1) $$q_{t}=\frac{\left(C_{0}-C_{t}\right)v_{0}}{m}$$

where *C*_0_ is the initial concentration of the solution (mg/L); *C_t_* is the concentration of the solutions at the time (*t*); *v*_0_ is the volume of the solution (mL); and m is the mass of the adsorbent (g).

## 3. Result and Discussion

**Figure 3:**
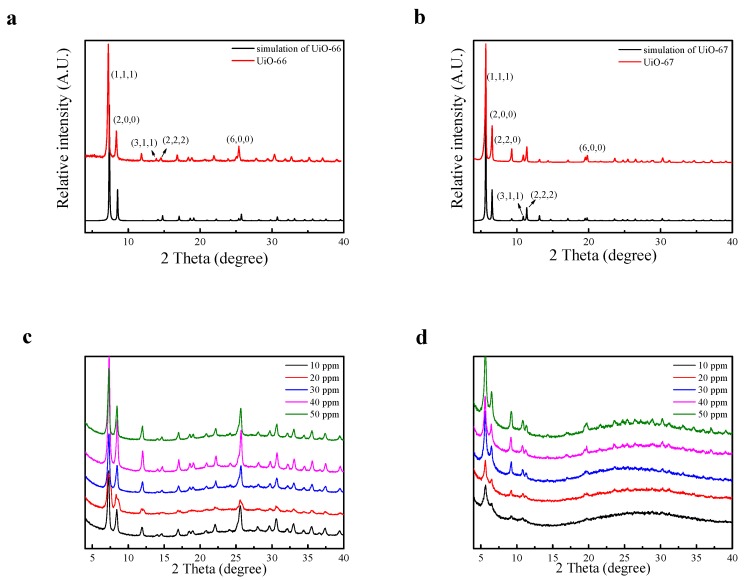
Powder X-ray diffraction (PXRD) patterns of (**a**) UiO-66, (**b**) UiO-67, (**c**) UiO-66 after adsorption, and (**d**) UiO-67 after adsorption.

In this work, HCl was introduced into the system as a modulator to speed up the reaction. Because of this, the reaction condition was much milder than that in a previous work [33]. Powder X-ray diffraction (PXRD) measurements of the samples were carried out to determine whether the structures of the UiOs were correct. As can be seen in Figure 3a, there were characteristic peaks at 7.2°, 8.5°, 14.1°, 14.7°, and 25.5°, which respectively corresponded to the (111), (200), (311), (222), and (600) lattice planes. As can be seen in Figure 3b, those characteristic peaks were at 5.6°, 6.5°, 9.3°, 10.9°, 11.3°, and 19.8°, which respectively corresponded to the (111), (200), (311), (222), and (600) lattice planes. Combining the PXRD data analyses of UiO-66 and UiO-67, it was possible to determine the similarities between UiO-66 and UiO-67 and their structural diagrams (Figure 1). Clearly, the positions of the diffraction peaks of UiO-66 and UiO-67 corresponded well with the simulated patterns, and no impurity peaks were observed, thus proving the high purity of the products (Figure 3). The diffraction peaks of UiO-66 and UiO-67 were sharp and intense, indicating that they were highly crystalline. To study the stability of the UiOs, samples of the UiOs before and after adsorption were collected and contrasted using PXRD. The PXRD patterns of the UiOs are shown in Figure 3. As shown in Figure 3a,b, the diffraction curves of the as-synthesized UiOs were visible and in good agreement with the simulation, demonstrating symmetric cubic structures and high crystallinity. As shown in Figure 3c,d, compared with the original materials, the UiOs after adsorption had no significant changes in peak positions, revealing the stability of the structure of the UiOs after the adsorption of AO7.

**Figure 4:**
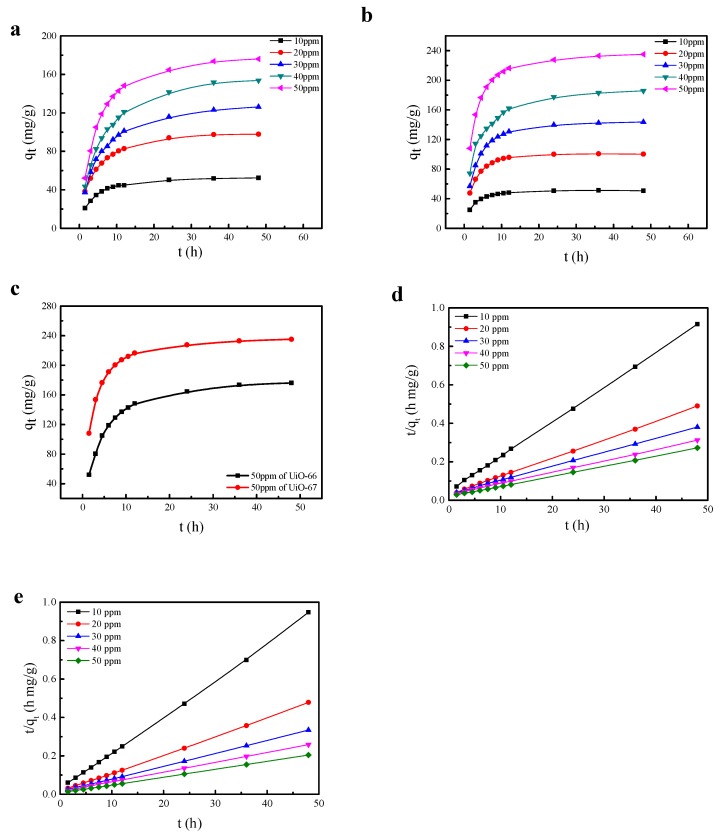
Effect of contact time and initial concentration of the AO7 aqueous solutions on the adsorptive capacity. (**a**) UiO-66, (**b**) UiO-67, and (**c**) UiO-66 and UiO-67 comparison of the maximum adsorption. Kinetic plots of *t*/*q_t_* vs. *t* for (**d**) UiO-66, (**e**) UiO-67.

Figure 4 indicated the variation of the adsorption capacity with time. The amount of adsorption gradually increased as a function of time. Furthermore, we noted that the larger the initial concentration of AO7, the greater the amount of adsorption. Table 4 shows the excellent adsorption capacities of UiO-66 and UiO-67, which are much greater than other materials [1,7,45,46]. Compared with natural materials, the Zr-MOFs in this work have considerable advantages in adsorption capacity. Even if natural materials are modified, their adsorption capacities will not achieve similar results to the MOFs. The adsorption capacity of the Zr-MOF is also stronger than that of graphene. Moreover, as can be seen in Figure 4c, the greatest adsorption capacity of UiO-67 is also superior to that of UiO-66. 

To understand the potential reaction mechanism, the changes in adsorptive capacity over time were simulated by theoretical kinetic models. From the reported literature, we can determine that in the initial concentration range of the experiment, the process of the UiOs adsorbing AO7 was in accordance with a pseudo-second-order kinetics model, and the adsorption model conformed to the Langmuir model [47]. Therefore, in this study, only the pseudo-second-order kinetics model was studied [48]. The pseudo-second order kinetic models were described by the following linear equation:

(2) $$\frac{t}{q_{t}}=\frac{1}{k_{2}q_{e}^{2}}+\frac{t}{q_{e}}$$

where *t* is the contact time (h), and *k*_2_ is the rate constant of the pseudo-second-order kinetic models.

The fittings of the pseudo-second-order kinetics are shown in Figure 4d,e, and the relevant parameters are listed in Table 1 and Table 2. The calculated kinetic constants (*k*_2_) decreased as the dye concentration increased, which was similar to cases of other dyes adsorbed by MOFs in the literature [44]. Under the circumstance of 10 and 20 ppm, *k*_2_ of UiO-67 was similar to that of UiO-66. From 30 to 50 ppm, *k*_2_ of UiO-67 was significantly less than that of UiO-66. Consequently, more dye molecules were adsorbed on the surface of the MOFs with the increase in the concentration, and the adsorption quantity of UiO-67 was greater than that of UiO-66. The preliminary conclusion here is that the bigger the pore size, the stronger the adsorption capacity, which can be verified by the N_2_ adsorption–desorption isotherms.

**Figure 5:**
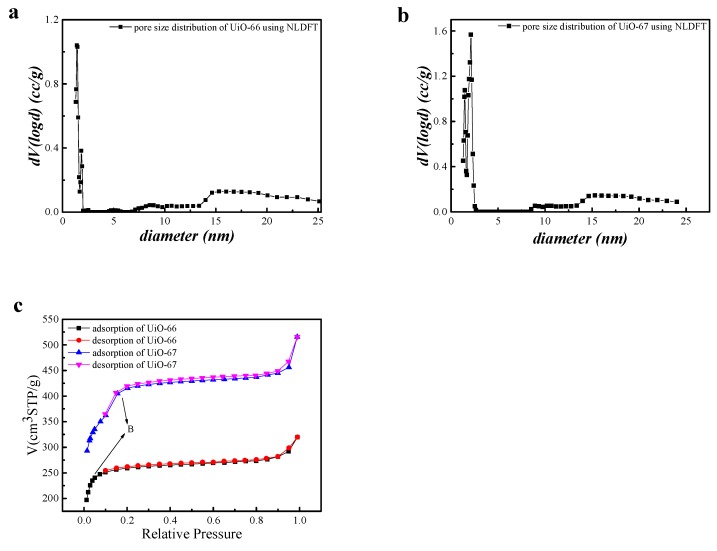
Pore size distribution desorption of (**a**) UiO-66, (**b**) UiO-67, and (**c**) isothermal N_2_ adsorption of UiO-66 and UiO-67.

The BET method [49,50,51] was utilized to estimate the specific surface area, the pore size, and the pore distribution. As can be seen in Figure 5, the pore size distributions of the Zr-MOFs suggested that UiO-66 was mainly microporous (1.4 nm) and UiO-67 was mainly mesoporous (2.1 nm). We can conclude from Figure 5c that the UiOs showed typical type I adsorption–desorption isotherms, which can be explained by a Brunauer–Emmett–Teller (BET) adsorption process [22]. Due to the fact that the pores are rapidly filled at very low relative pressures (<0.01), point B is usually regarded as a sign of the end of the monolayer adsorption capacity and is usually followed by multilayer adsorption [52]. With respect to the characteristics of physical adsorption and chemical adsorption, we can determine that chemical adsorption is a single-layer adsorption, in which the speed is slow, while physical adsorption is multilayer adsorption, in which the speed of adsorption is fast. In the low P/P_0_ region, the curve was convex upwards, which reflected the strong interaction between the adsorbate and adsorbent. 

Taken together, the adsorptive behavior of the UiOs was dictated by both chemisorption and physisorption phenomena. As illustrated in Table 3, which shows the degree of porosity evaluated using non-local density function theory (NLDFT) and Barrett, Joyner, and Halenda (BJH) analysis, we can conclude that the pore width using the NLDFT method was more consistent with the theoretical value. NLDFT, considered as an accurate calculation method for mesoporous materials, was adapted to calculate the specific surface area of the UiOs and provided a more precise analysis of the pore structure of the UiOs [53,54]. From the calculation results of NLDFT, we can conclude that the degree of porosity of UiO-67 was larger than that of UiO-66.

As shown in Figure 4a,b, by comparing the curves, we determined that the adsorption process of the UiOs is initially rapid and then tends to become gentle. UiO-66 and UiO-67 have the same adsorption capacity for dyes in the range of low dye concentration. Nevertheless, in the range of high dye concentration, the higher the concentration, the more advanced the line of diffusion, and the stronger the reaction of the dye molecules to the materials. In the latter stage of adsorption, the adsorption curve tends to be stable, which is due to the fact that the UiOs undergo a single-layer reversible adsorption. Combined with the analysis of the nature of the adsorption of the UiOs, we can conclude that the UiOs’ adsorption of the dye molecules was mainly carried out by physical multilayer adsorption, accompanied by chemical single-layer adsorption. In contrast to the adsorption of other materials on AO7, UiOs have obvious advantages which shown in Table 4.

In the whole adsorption process, physical adsorption takes the dominant position, which can be considered as natural diffusion. From the mechanism of adsorption, we can conclude that the multilayer adsorption of mesoporous materials mainly occurs on the surface of the materials, and chemical adsorption is a hole-filling process of micropores [22]. Owing to the pore size of UiO-67 being larger than that of UiO-66, UiO-67 is superior to UiO-66 with respect to the adsorption of the dyes. On account of the fact that physical adsorption takes place on the surface of the material, the adsorption capacity depends not only on the specific surface area, but also the amount of the dye. This point can be verified by the phenomenon of the UiOs adsorbing high concentrations of the dye. AO7 is a kind of azo dye, the structure of which can be seen in Figure 2. The UiOs are composed of metal–oxygen clusters (Zr_6_O_4_(OH)_4_) and ligand, the structure of which can be seen in Figure 1. Through a comparison of the structures of the UiOs and AO7, we can conclude that the chemical adsorption of AO7 by the UiOs mainly depends on the metal–oxygen clusters. As mentioned previously, the structural difference between UiO-66 and UiO-67 is mainly determined by the difference in ligands. Moreover, the UiOs’ adsorption capacity of the dye depended on the size of the pores. In conclusion, the larger the pore size, the stronger the adsorption capacity of the materials.

## 4. Conclusions

In summary, we have reported the synthesis of Zr-MOFs with hydrochloric acid as the accelerator, which can serve as an efficient method to obtain different apertures of UiO-66 and UiO-67, composed of different lengths of organic ligands in milder conditions (80 °C). N_2_ adsorption–desorption isotherms provided effective evidence that the aperture of UiO-66 was smaller than that of UiO-67. Moreover, we have analyzed the adsorption mechanisms of the Zr-MOFs on AO7 with Zr-MOF structure and adsorption dynamics models. The adsorption behavior of the Zr-MOFs on the dye molecules was mainly performed by natural diffusion and was accompanied by chemical adsorption. The natural adsorption was the multilayer adsorption that occurred on the material’s surface, and the chemical adsorption occurred in the pores where the dye molecules combined with the active sites in the form of Langmuir monolayer adsorption. Due to the fact that the structural differences between UiO-66 and UiO-67 are reflected in the lengths of their ligands, physical adsorption plays a leading role in the adsorption of the dye by a Zr-MOF. Moreover, a Zr-MOF’s capacity to adsorb dye depends on the size of the pores. We concluded that the larger the pore size, the stronger the adsorption capacity of the materials.

## Figures and Tables

**Table 1:** Parameters of pseudo-second-order kinetics model for the adsorption of AO7 by UiO-66 at series initial concentrations.

*C*_0_ (ppm)	*k*_2,ad_ (g/mg h)	*q_e_* (mg/g)	*R* ^2^
10	0.01803	56.21	0.99986
20	0.00954	104.57	0.99965
30	0.00729	132.40	0.99984
40	0.00589	161.99	0.99966
50	0.00525	189.54	0.99973

**Table 2:** Parameters of pseudo-second-order kinetics model for the adsorption of AO7 by UiO-67 at series initial concentrations.

*C*_0_ (ppm)	*k*_2,ad_ (g/mg h)	*q_e_* (mg/g)	*R* ^2^
10	0.01897	55.89	0.99949
20	0.00960	110.89	0.99952
30	0.00664	156.62	0.99977
40	0.00511	196.76	0.9998
50	0.00410	253.09	0.99994

**Table 3:** The degree of porosity evaluated using non-local density function theory (NLDFT) and Barrett, Joyner, and Halenda (BJH) analysis.

	UiO-66 (NLDFT)	UiO-66 (BJH)	UiO-67 (NLDFT)	UiO-67 (BJH)
**Surface Area (cm^3^/g)**	744	793	1139	1314
**Pore Width (nm)**	1.410	30	2.114	30
**Pore Volume (cc/g)**	0.413	0.093	0.649	0.146

**Table 4:** The comparison of the UiOs’ adsorption of AO7 with the other reported adsorbents.

Adsorbents	*Q*_max_ (mg/g)
Grapheme-cetyltrimethy-lammonium bromide (GN-CTAB) [1]	146
Modified Rice Stem [46]	38
Coconut Coir [45]	14
Natural pumice (NP) [7]	15
Fe–CP [7]	27
UiO-66 (This Work)	176
UiO-67 (This Work)	235
